# 

**Published:** 1884-08

**Authors:** 


### BOOKS AND PAMPHLETS RECEIVED.

Hooper's Physicians' Vade Mecum—A manual of the principles and practice of physic, with an outline of general pathology, therapeutics and hygiene. Tenth edition revised by William Augustus Guy, M. B. Cantab., F. R. S., and John Harley, M. D., London, F. L. S., vol. ii, Wm. Wood & Co., 56 and 58 LaFayette Place, New York, 1884.

Quarantine and Sanitary Operations of the Board of Health of the State of Louisana during 1880, 1882, 1883 and 1884, by Joseph Jones, M. D., President of the Board of Health of the State of Louisiana, 1884.

AUSCULTATION, PERCUSSION AND URINALYSIS, by C. Henri Leonard, M. A., M. D. The Illustrated Medical Journal Company, publishers, 1884.

SOUTHSIDE VIEWS—Dr. Whedon and the Fathers; also Dr. Haygood's Our Brother in Black, by Rev. W. J. Scott, North Georgia Conference. Jas. P. Harrison & Co., publishers, Atlanta, 1884.

Student's Manual of Electro-therapeutics, embodying lectures delivered in the course on therapeutics, at the Woman's Medical College of the New York Infirmary, by R W. Amidon, A. M., M. D. G. P. Putnam's Sons, publishers, New York, 1884.

MEMOIR ON THE NATURE OF DIPHTHERIA, by Drs. H. C. Wood and H. F. Formad, Philadelphia, Appendix A, report of the National Board of Health, 1882. J. B. Lippincott & Co., publishers, Philadelphia, 1884.

EDUCATION the means for unifying the medical profession and strengthening the State Medical Association, by R. A. Kinloch, M. D., President of the South Carolina Medical Association. Reprinted from the Transactions of the South Carolina Medical Association 1884, by special resolution.

CONGENITAL LIPOMA, by A. Jacobs, M. D., Clinical Professor of Diseases of Children, College of Physicians and Surgeons, New York. Reprinted from the Archives of Pediatrics, vol. 1. No. 11, 1884.

Strictures of the Œsophagus, their Nature and Treatment, with Cases, by Henry F. Campbell, M. D., Augusta, Ga. Extracted from the Transactions of the American Surgical Association, vol. 1, 1883.

On Paroxysmal Fever, not Malarial, by J. H. Masser, M. D., Physician in charge of the Medical Dispensary of the Hospital of the University of Pennsylvania, Pathologist to the Presbyterian Hospital. Reprinted from the proceedings of the Ph ladelphia County Medical Society, 1884.

Circulars of Information of the Bureau of Education No. 3, 1884. Government Printing Office, Washington, D. C.

Criminal Responsibility of the Insane. by Orpheus Everts, M. D., Medical Superintendent Cincinnati Sanitarium, College Hill, Ohio.

How to Grow Fine Celery—a new method, by Mrs. H. M. Crider. Price 25 cents. H. M. Crider, Publisher, York, Pa., 1884.

Forty-Eighth Annual Announcement of the Medical Department of the University of Louisville, session 1884-'85.

The New York Post-Graduate Medical School and Hospital Announcement of the third year. Published for the Faculty by J. H. Vail & Co., New York. 1884.

### OUR ADVERTISERS.

*The Atlanta Medical College.*—This old and popular institution has an advertisement in the JOURNAL. Improvements are now being made which will be complete by the first of October next, which will render the hospital advantages equal to any school in the South. See the advertisement and write the Proctor for the annual announcement.

*The New York Pharmacal Association.*—This Association manufactures the best digestive agent in the market. We refer to Lactopeptine. We have used it extensively for several years, and can confidently recommend it as a valuable agent in summer diarrhœa of children.

*The Jefferson Medical College, Philadelphia.*—This popular college is too well known to need any words of commendation from us. The next session will begin October 1st, 1884, and close March 31st, 1885. The advertisement will be found elsewhere in the JOURNAL.

*Miami Medical College, Cincinnati.*—The regular session of this college will begin September 18th and continue five months. This school presents unusual clinical advantages to students. Besides the daily clinics in the college dispensary, the students have access to the great Cincinnati Hospital, which affords unusual advantages for studying diseases of all kinds. See the advertisement and write the Dean for the annual announcement.

*Jacobs' Pharmacy, Atlanta*—This is a new enterprise for Atlanta. Mr. Jacobs came here in January, and has already established a fine business. He makes a specialty of manufacturing fluid extracts from indigenous drugs, such as stillinga, gelsemium, phytolaca, etc. These fluid extracts are all made from the green roots, and must be very greatly superior to preparations made from the dried roots, for it is a well known fact that certain volatile active principles of the drugs are lost when allowed to dry. When they are made from the green roots they contain all the therapeutic virtues of the drug. Mr. Jacobs has agents employed who understand how and when to gather the roots that these extracts are made from. He has been manufacturing these goods for five years in Athens, and came to Atlanta in order that he might enlarge his business. He makes a specialty of McDade's formula, as he received it from the late Dr. Marion Sims. See his advertisement and write him for catalogue of his new preparations.

*Reed & Carnrick, New York.*—These gentlemen manufacture the justly celebrated Beef Peptonoids. This is a preparation of the dry extract of beef with an equal portion of gluten. It was used with great advantage in the treatment of the late President Garfield. We have found it especially applicable in convalescence from fevers and can confidently recommend it as a valuable preparation.

*McBride & Co., Atlanta.*—We present the advertisement of this reliable house in another part of the JOURNAL. They are proprietors of the celebrated Gate City Stone Filter, the best filter we have ever seen. The managing editor of the JOURNAL has been using one of them for two years and can unhesitatingly recommend it as a superior filter.

*Iron Dyed Surgeon's Silk.*—This excellent preparation of surgeon's silk is on sale at the drug store of Chas. O. Tyner, at the corner of Marietta and Broad streets, Atlanta. It is superior to any silk ligature we ever used.

*A New Institution.*—Drs. Wm. Abram and Thos. D. Love have recently established a new Infirmary in this city for the treatment of diseases of women and children. The reputation of Dr. Wm. Abram Love is too well known to need any words of commendation from us. His extensive practice in this specialty renders him peculiarly fitted to be in charge of such an institution. Dr. Thos. D. Love, his assistant, is a young man, but none the less competent. See their advertisement, to be found elsewhere in the JOURNAL.

*The Georgia Pacific Railway.* – Among the great highways of travel which now carry the tourist, pleasure seeker, business man or emigrant to New Orleans, Memphis and the West, none have risen superior to the Georgia Pacific Railway in appreciation of the requirements of modern railway travel. All that energy and skill, combined with capital, could do, has been done in giving safety combined with speed and providing the luxurious and elegant appointments which the traveling public demand who travel for enjoyment. Appreciating safety above all other requirements, especial attention has been bestowed upon the railway. Heavy steel rails, stone ballasted track and iron bridges all combine to insure the success that was striven for, and the record of train service in regularity and freedom from accident will bear a favorable comparison with any railway on the continent. All of the most improved appliances of train equipments: automatic air-brakes, Miller platforms and Janney couplers have been provided. The motive power consists of the largest locomotives in the South, built especially for this important thoroughfare to haul the fast New Orleans express trains, which leave Atlanta daily, connecting at New Orleans with the Texas railroad for the West. Parties contemplating taking a trip to Mississippi, Louisiana and the West will find it to their advantage to write to Mr. Alex. S. Thweatt, Traveling Passenger Agent of the Georgia Pacific Railway, Atlanta, Georgia, who will take pleasure in furnishing you with any information desired.

### CONTENTS.

ORIGINAL COMMUNICATIONS—

### NOTICE.

Original communications solicited. When articles require illustration the engravings will be furnished by the publishers free of charge.

Subscription: $2.50 a year.

Subscribers will please not send us money by Postal Notes. Remit by Express, Draft, Post-office Order, or Registered Letter.

Address all letters and books, and make all drafts payable to

The Atlanta Medical and Surgical Journal,
Care Jas. P. Harrison & Co., Atlanta, Ga.

